# Design of a trial to evaluate the impact of clinical pharmacists and community health promoters working with African-Americans and Latinos with Diabetes

**DOI:** 10.1186/1471-2458-12-891

**Published:** 2012-10-23

**Authors:** Ben S Gerber, Lauren Rapacki, Amparo Castillo, Jessica Tilton, Daniel R Touchette, Dan Mihailescu, Michael L Berbaum, Lisa K Sharp

**Affiliations:** 1 Institute for Health Research and Policy, 1747 West Roosevelt Rd. M/C 275, Chicago, IL, 60608, USA; 2 Midwest Latino Health Research, Training and Policy Center, 1640 West Roosevelt Road- Suite 636, Chicago, IL, 60608, USA; 3 Department of Pharmacy Practice, 833 S. Wood St. M/C 886, Chicago, IL, 60612, USA; 4 Section of Endocrinology, Diabetes and Metabolism, 1819 West Polk Street, M/C 640, Chicago, IL, 60612, USA; 5 Jesse Brown VA Medical Center, 820 South Damen Ave., Chicago, IL, 60612, USA

**Keywords:** (3–10): Diabetes mellitus/drug therapy, Patient compliance, Patient education, Pharmacists, Community health workers

## Abstract

**Background:**

Given the increasing prevalence of diabetes and the lack of patients reaching recommended therapeutic goals, novel models of team-based care are emerging. These teams typically include a combination of physicians, nurses, case managers, pharmacists, and community-based peer health promoters (HPs). Recent evidence supports the role of pharmacists in diabetes management to improve glycemic control, as they offer expertise in medication management with the ability to collaboratively intensify therapy. However, few studies of pharmacy-based models of care have focused on low income, minority populations that are most in need of intervention. Alternatively, HP interventions have focused largely upon low income minority groups, addressing their unique psychosocial and environmental challenges in diabetes self-care. This study will evaluate the impact of HPs as a complement to pharmacist management in a randomized controlled trial.

**Methods/Design:**

The primary aim of this randomized trial is to evaluate the effectiveness of clinical pharmacists and HPs on diabetes behaviors (including healthy eating, physical activity, and medication adherence), hemoglobin A1c, blood pressure, and LDL-cholesterol levels. A total of 300 minority patients with uncontrolled diabetes from the University of Illinois Medical Center ambulatory network in Chicago will be randomized to either pharmacist management alone, or pharmacist management plus HP support. After one year, the pharmacist-only group will be intensified by the addition of HP support and maintenance will be assessed by phasing out HP support from the pharmacist plus HP group (crossover design). Outcomes will be evaluated at baseline, 6, 12, and 24 months. In addition, program and healthcare utilization data will be incorporated into cost and cost-effectiveness evaluations of pharmacist management with and without HP support.

**Discussion:**

The study will evaluate an innovative, integrated approach to chronic disease management in minorities with poorly controlled diabetes. The approach is comprised of clinic-based pharmacists and community-based health promoters collaborating together. They will target patient-level factors (e.g., lack of adherence to lifestyle modification and medications) and provider-level factors (e.g., clinical inertia) that contribute to poor clinical outcomes in diabetes. Importantly, the study design and analytic approach will help determine the differential and combined impact of adherence to lifestyle changes, medication, and intensification on clinical outcomes.

**Trial registration:**

ClinicalTrials.gov identifier: NCT01498159

## Background

Individuals with diabetes are at greater risk for cardiovascular disease, the leading cause of death among men and women [1]. To reduce the risk of cardiovascular disease, attentive management of blood glucose, blood pressure, and LDL-cholesterol levels is paramount. Despite available treatments, less than 10-20% of people achieve their goals in therapy [2-7]. While time-trend analyses show improvement in blood glucose, blood pressure, and cholesterol control, African-Americans and Latinos have worse control of these risk factors compared with Whites [8-13]. These differences in intermediate factors may contribute to higher complication rates in minority groups, such as renal disease, coronary artery disease, and amputations, as well as mortality [9,14,15].

In order for most individuals to reach their goals in therapy, regular, ongoing adherence to medication is required. Common factors that contribute to non-adherence include low health literacy, depression, lack of medication knowledge, lack of social support, concerns about adverse events and dependency, poor patient-clinician communication, and limited access to healthcare resources [16,17]. In the Latino population, additional barriers may include low English proficiency, cultural issues, and poorer access to conventional healthcare. Additionally, low adherence may reduce treatment modifications and intensification [18,19].

From the healthcare provider perspective, intensification of medication therapy is often limited by “clinical inertia,” which occurs when providers fail to appropriately intensify therapy in patients despite recognizing elevations in blood glucose, blood pressure, or cholesterol [20]. Clinical inertia is sometimes related to providers having limited time allotted for each encounter and patients presenting with concerns that compete for attention [20]. In addition, providers are often not trained in the complex array of psychosocial or environmental factors that affect adherence which must be addressed prior to intensification of medication [21].

Given the increasing prevalence of diabetes and the lack of patients reaching recommended therapeutic goals, novel models of team-based care are emerging. These teams typically include physicians, nurses, case managers, pharmacists, and community-based peer health promoters (HPs) [17,22-24]. Recent evidence supports the role of office-based clinical pharmacists in diabetes management to improve glycemic control, as they offer expertise in medication management with the ability to collaboratively intensify therapy [25-27]. Such pharmacists are often found in primary care teams providing diabetes management [28,29].

Community-based health promoters may further enhance the effectiveness of clinical pharmacists in medication management [17]. Health promoter interventions have focused largely upon low income minority groups, addressing their unique psychosocial and environmental challenges in diabetes self-care. Health promoters can perform home visits and telephone calls to provide education, evaluate medication use, promote behavioral change and self-management, and reinforce pharmacist and other provider recommendations. Such measures may improve medication adherence and support intensification to reach goals in blood sugar, blood pressure, and cholesterol.

Building upon a pilot study, the present study was designed to determine the effectiveness of pharmacist-health promoter team management in addressing medication adherence and intensification [17]. In addition, the study will investigate the benefit derived from adding HP support following receipt of clinical pharmacist services, and maintenance of improved behaviors and outcomes after phasing out health promoter support. Finally, cost and cost-effectiveness of this team approach will be examined.

## Materials/Design

### Objectives

Our primary goal is to evaluate the effectiveness of pharmacist plus health promoter support (*Pharmacist+HP*) compared with pharmacist alone (*Pharmacist*) on diabetes behaviors (including healthy eating, physical activity, and medication adherence), hemoglobin A1c, blood pressure, and LDL-cholesterol levels among African-American and Latino adults with uncontrolled type 2 diabetes. Secondarily, we will evaluate the maintenance of improved diabetes behaviors, as well as clinical outcomes, by phasing out health promoter support from the *Pharmacist+HP* group after one year. Also, we will evaluate the intensification offered by adding a health promoter after one year of Pharmacist alone. Finally, we will determine the cost and cost-effectiveness of the *Pharmacist+HP* and *Pharmacist* alone approaches.

### Study design

**Figure 1:**
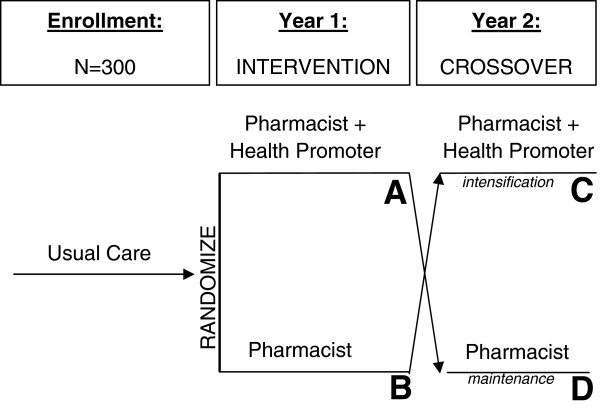
**Study Design.** The proposed study is a randomized trial with crossover after one year. Three hundred African-American and Latino patients with uncontrolled type 2 diabetes will be randomized to receive either *Pharmacist* or *Pharmacist+HP* conditions. After one year, groups will crossover. The *Pharmacist* group will be intensified by adding health promoter support. Concurrently, maintenance will be evaluated in the *Pharmacist+HP* group by phasing out health promoter support.

The proposed study is a randomized trial with crossover after one year (Figure 1). Three hundred African-American and Latino patients with uncontrolled type 2 diabetes will be randomized to receive either:

1. *Pharmacist*: Clinical pharmacists will provide medication and disease management services to patients following a Pharmacist Management Protocol.

2. *Pharmacist+HP*: Includes clinical pharmacist services, plus a health promoter who will attend to both lifestyle and medication adherence-related issues by identifying barriers, solving problems, and providing support through home visits and telephone contact.

After one year, groups will crossover. The *Pharmacist* group will be intensified by adding health promoter support. Concurrently, maintenance will be evaluated in the *Pharmacist+HP* group by phasing out health promoter support. Outcomes in terms of medication adherence, A1c, blood pressure, and LDL-cholesterol levels will be evaluated at 0, 6-, 12- and 24-months.

### Ethics

The research protocol has been approved by the University of Illinois at Chicago Institutional Review Board (2011–0099).

### Study population and site

The University of Illinois Medical Center (UIMC) includes both inpatient and outpatient facilities serving a diverse, low-income population in Chicago. There are approximately 8,000 unique African American or Latino individuals receiving care for diabetes in the outpatient setting annually. The UIMC has multiple off-site ambulatory centers staffed by Family Practice and Internal Medicine primary care physicians (PCPs), and include affiliated Federally Qualified Health Centers (FQHCs). We anticipate participation of 6–8 clinical entities in the study. All sites share access to the electronic medical record (EMR), Cerner Powerchart (Cerner Corporation, Kansas City, MO).

### Inclusion criteria

Patients will be eligible for the study if they: (a) are self-identified as Latino/Hispanic or African-American; (b) have verbal fluency in English or Spanish; (c) are age 21 or above; (d) have a history of type 2 diabetes; (e) have an elevated A1c level 8% or higher in the past year (confirmed through EMR); (f) received primary care at UIMC for at least one year; and (g) take at least one oral medication for diabetes or hypertension (for adherence assessment).

### Exclusion criteria

Patients will be excluded from the study if they: (a) are unable to verbalize comprehension of study or have evidence of impaired decision making (e.g., dementia); (b) live outside of the Chicago area three or more months of the year; (c) have a household member already participating in same study; (d) plan to move from the Chicago area within the next year; or (e) are pregnant or trying to become pregnant.

### Identification, recruitment, and randomization of participants

The recruitment process will be carried out separately for each ambulatory site as determined by the project coordinator and clinical staff. On-site research assistants (RAs) will receive referrals from staff or patients interested in being screened for the study. All participant medical records are reviewed by a study physician to ensure A1c eligibility. Individuals deemed eligible will be scheduled for an appointment at the university Clinical Interface Core facility to complete written consent, HIPAA authorization, and baseline data collection. The RAs will inform patients that the research is being conducted to improve medication management in diabetes care and to find out whether pharmacists and health promoters help people reach goals of therapy. The RAs will spend additional time discussing the study protocol in detail as part of consent procedures and use the “teach-back” method to ensure participant comprehension [30].

Randomization to receive either *Pharmacist* or *Pharmacist+HP* assignments for the first year will occur after consent and completion of all baseline data collection. A random sequence of 300 participant assignments will be used. Blocked randomization will ensure that allocation to experimental conditions will be balanced within four gender by ethnicity subgroups at each site. The project coordinator will independently determine group assignments and will log the information separately. Baseline results for A1c and fasting lipid profile are entered into the patient’s medical record by a study physician and electronically shared with the primary care physician and pharmacist.

### Sample size calculation

Sample size calculations are powered to detect change in A1c. Based on a previous study [31], we estimated a mean baseline A1c level of 10.0% with standard deviation of 1.8% and an effect size of (1.0/1.8) 0.56. The cross-time correlation was estimated to be 0.30. We adjusted for clustering, assuming an intraclass correlation coefficient (ICC) of 0.02 and cluster size of 30, which yields a design effect of 1.58. Two-sided alpha of 0.05 and 80% power are assumed requiring a minimum of 114 per group. Allowing for an estimated 10% drop-out during each of the two years, approximately 150 patients will be required for each group (or 300 total).

### Data analyses

Data will be analyzed for clinical effectiveness using intention-to-treat principles [32-34]. To represent dropouts in the analysis, missing data will be imputed using various approaches, such as “last value carried forward.” The Little and Yau “return to baseline” imputation rule, in which any gains over baseline levels dissipate upon dropout, will be used for primary analyses. Patients who share a single primary care physician (PCP) or health promoter (HP), might exhibit similarities that are not shared with patients attended to by other physicians. To address this, we will include random effects in the model for clinic site, PCP, and HP. We will include as predictors relevant care and treatment-related variability such as medication therapy intensifications and clinical encounter frequency.

We will test the hypothesis that A1c, blood pressure, and LDL-cholesterol levels will be lower and diabetes behaviors (including medication adherence) will be improved with patients receiving *Pharmacist+HP* compared with those receiving *Pharmacist* alone. Univariate comparisons between the two study groups with respect to outcome measures and covariates at baseline will be conducted using Chi-square tests for categorical variables, Kruskal-Wallis tests for ordinal variables, and linear mixed models for continuous variables. Non-normal continuous data will be transformed prior to linear model analyses. The baseline variables include demographic, therapy complexity, and health literacy data. If we find any between-group differences at baseline, we will adjust for this in our primary multivariate analyses by including relevant covariates.

We will also extend the usual analysis of crossover designs by including a longitudinal trend component in the first year. Thus, we can examine the time course (0, 6, and 12 months) as linear or quadratic over the first three measurements. This will allow us to investigate whether changes are made early, and at what rate they continue through the rest of the time period. This analysis permits the comparison of trends between *Pharmacist* and *Pharmacist+HP* conditions. In addition, we will regard participants as a random effect and will use Gaussian mixed-model estimations (SPSS MIXED command). We can then substitute treatment by period interactions for the carry-over effects, and the model can be reduced in a recommended sequence (first omit carry-over, then omit treatment, finally omit period).

The primary analysis of each outcome in the repeated measures design will be conducted in a general linear mixed model framework. In addition, SPSS MANOVA (for repeated measures) will be used to explore the simultaneous impact of the treatment on multiple correlated dependent variables, including the use of Roy-Bargmann stepdown F-tests and discriminant function analysis as post hoc tests to identify subsets of outcome measures affected [35]. SPSS MIXED will be used to explore patterned covariance structures such as compound symmetry and autoregression along with incorporating time-varying covariates, such as depression. MANOVA secondary analyses will explore the impact on results of inhomogeneous baseline variables. Exploratory subgroup analyses will determine which participants in the intervention group had the greatest improvement in outcomes, based on demographic factors, health literacy, and medication regimen. Group by time-trend interaction contrasts also will be used to explore different group trajectories of change. In addition, we will investigate the potential consequences of medication intensification (e.g., initiation of insulin) by evaluating changes in body mass index and severe hypoglycemia.

We will explore diabetes-related behaviors (including eating habits, physical activity, and medication adherence) as well as medication treatment intensification as alternative mediators using MPlus [36]. We will also test the hypothesis that diabetes behaviors and clinical outcomes will be maintained one year after phase-out of HP support. Finally, we will test the hypothesis that diabetes behaviors and clinical outcomes improved by adding HP support after one year of *Pharmacist* alone.

### Pharmacists

Pharmacists will provide medication and disease management services to patients following an established Pharmacist Management Protocol. In providing care, pharmacist disease management services are comparable to other disease management programs and include comprehensive needs assessments, proactive health promotion, patient-centric goals and education, interventions to encourage behavioral change, and PCP support and feedback [37]. The pharmacists will review current medication use, identify therapeutic goals (particularly A1c, blood pressure, and LDL cholesterol), formulate a PCP-approved plan of care, and document the plan in the EMR. Pharmacists will educate and encourage lifestyle changes based upon ADA nutrition and physical activity guidelines [38,39]. All patients recruited into this study will require medication to control their diabetes. The PCPs and pharmacists will decide on the algorithm and approach to intensify therapy and how medication changes may be made (based on national guidelines) [40-42]. The intention is to establish optimal communication with minimal PCP burden (a similar approach to our pilot study). Individual goals will be identified for each participant for A1c, blood pressure, and LDL-cholesterol (e.g., A1c ≤ 7%, blood pressure < 130/80 mm Hg, and LDL < 100 mg/dL). In the proposed study, pharmacists will adopt the ADA approach to individualized care, where the general goal for non-pregnant adults is A1c less than 7%. The pharmacists may decide upon less stringent goals for those with a history of severe hypoglycemia, limited life expectancy, advanced micro- or macrovascular disease, or extensive comorbid conditions [43].

Initially, the pharmacists will meet with patients to reconcile medications and discuss therapeutic goals. Next, the pharmacists will assess common barriers to medication adherence including poor memory, lack of diabetes knowledge, health beliefs, cost, medication burden, physical disabilities, and social barriers. Following the initial visit, encounters may occur in person, at the clinic, or by phone. Duration between encounters may increase based on individual preference and when participants reach goals (with maximum of three months). The pharmacist activities will include an evaluation of adherence, medication reconciliation, and review of home glucose and/or blood pressure monitoring log data. Pharmacist education of patients will target medication (name and purpose of medications; time, strength, and method of administration); drug interactions and side effects; goals of therapy; basic lifestyle modifications; and use of pillbox, low-literacy medication lists, or other adherence aids.

Pharmacists will adjust therapy according to the plan of care under PCP guidance and notify PCPs of agreed-upon modifications via the EMR. Side effects identified by the HP or pharmacist will be conveyed to the PCP immediately and if the PCP is not available, the covering clinic physician will be contacted per Pharmacist Management Protocol. Laboratory assessments including electrolytes and renal function will be completed per medication titration protocol. In addition to scheduled study-related clinical data collection, point of care testing will be performed during pharmacist visits as needed based on guidelines to further guide treatment plans. For hypoglycemia, pharmacists routinely monitor hypoglycemic events, address prevention and review treatment. This includes three steps: (1) addressing hypoglycemia with every patient contact; (2) applying principles of aggressive therapy (education, empowerment, frequent glucose self-monitoring, flexible medication regimen, individualized goals, professional guidance); and (3) considering risk factors for hypoglycemia [44].

PCPs and clinical pharmacists are located in the same medical center building. The pharmacists have direct access to the PCP and routinely communicate in person, by telephone, and by EMR messaging depending on urgency. The pharmacist may adjust the medication regimen and provider refills in accordance to the PCP-directed care plan and response to therapy (to optimize blood glucose, blood pressure, and cholesterol levels). All adjustments, refills, and testing are completed under guidance of the participants’ providers and updated in the EMR medication list. Pharmacists have access to participants’ full medical records and can review flow-sheet and other chart data including blood test results, clinical progress notes, problem and medications lists, drug allergies, hospitalization records, and emergency room reports. Pharmacists will forward all electronic progress notes to the PCP EMR “Inbox” when medication changes are made. Progress notes include a detailed list of medications, estimated adherence levels, and home glucose and blood pressure monitoring log information. However, for urgent medical reasons, the PCP or covering provider will be contacted. If, for any reason, the PCP feels that the patient should not remain in the study, the provider can inform the PIs to disenroll the participant.

### Health promoters (HPs)

Health promoters conduct patient encounters, provide medication and lifestyle adherence support and communicate with pharmacists. Each clinical site will work with English- and Spanish-speaking health promoters (HPs) who are each responsible for up to 25 participants. Candidates for HP positions will be identified through advertisements, announcements, and organization meetings targeting these contacts. HPs will have: (1) U.S. citizenship; (2) a vehicle for transportation; (3) a bachelor’s degree; and (4) excellent communication skills. HPs will represent the communities being served.

All HPs will receive standardized training/re-training via two educational curricula: (1) the *Diabetes Empowerment Education Program* (DEEP) [45]; and (2) *Training Curriculum for Health Coaches*[46]. DEEP uses an empowerment/autonomy framework. In addition, it includes adult learning methodologies and interactive group exercises with role-playing. The program trains HPs in increasing patients’ knowledge, skills, and autonomy related to diabetes management and control. The training addresses physical activity, nutrition, psychosocial support, medication use, and the health care team interaction. The *Training Curriculum for Health Coaches*, includes sessions on the collaborative paradigm (*ask* instead of *tell*), action plans, problem solving, cardiovascular disease and medication management. HPs will also observe and shadow pharmacists’ interactions with patients in the UIMC Medication Therapy Management (MTM) Clinic as an apprenticeship to gain insight into clinical pharmacists’ roles and activities, such as medication reconciliation. Following training, we will assess HP competencies in counseling and diabetes-related clinical skills. HPs are video-recorded with standardized patients to provide evaluation and feedback on coaching and empowerment activities.

In addition, health promoter training will include time to orient HPs to resources available to patients in the medical facility (e.g., on-site pharmacy and social workers). There will be time allocated for the HPs to tour the medical campus to gain familiarity of the various clinical sites where they could meet with participants individually or jointly with pharmacists. Other training experiences include didactic sessions with a certified dietician (with trips to a grocery store to review food labels and compare products), clinical observations of health care providers counseling on medication use, and independent learning through video and computer multimedia. Periodic educational sessions are provided by specialists from the medical center.

HP encounters will include home visits and telephone contact. By performing home visits, the HPs may evaluate home issues related to lifestyle changes (e.g., food inventory), medication adherence (e.g., medication storage), and technique of injecting insulin and testing blood sugar. In addition, the HPs will have glucose meters and automated blood pressure home measurement devices to check blood pressure and blood sugar. The HP encounter documentation will include duration of encounter, topics of discussion, medications addressed, and need for pharmacist communication. The HPs and pharmacists will communicate in-person, by phone, or by secure e-mail to discuss participant issues.

HPs will attend to medication adherence-related issues by identifying barriers, solving problems, and providing autonomy support. For example, HPs will address language barriers with providers, limited health literacy, transportation barriers, and cultural barriers (e.g., alternative therapy use that may interfere with or replace the use of conventional medicines). HPs will attempt to elicit and address concerns, beliefs, and social norms that may threaten acceptance of and adherence to conventional therapies. The HPs will parallel pharmacist activities by evaluating adherence, assisting in medication reconciliation, reviewing home glucose and blood pressure monitoring data, and providing reinforcement of proper medication use. They will assist in the implementation of pillbox use and other adherence aids as needed. Also, HPs will have an iPad for participant use of multimedia for education, skill building, and motivation (*“Living Well with Diabetes”*) [31].

HPs will also provide education and support that reinforce lifestyle adherence in conjunction with medication adherence. HPs are trained on the diabetic diet along with basic physical activity recommendations so that they can work with patients to set individual goals. Education will address realistic and achievable food choices, portion sizes, cooking preparation, relationships between medications-meals-glucose levels, integration of physical activity into lifestyle, and local community resources for grocery shopping and physical activity.

### Study measures

Control variables will be completed at baseline as follows. *Socio-demographic Data* will include age, gender, self-reported race and ethnicity, country of origin (if other than U.S.), income, highest level of education, current employment status, marital status, number of adults and children at home (and adults with diabetes), global health status [47], and insurance. If the participant is Latino, data will also include language spoken and thought in, provider language spoken, and use of interpreter. *Diabetes and Medical History* will include time since diabetes diagnosis, receipt of diabetes education, home glucose monitoring frequency, and self-reported provider visit frequency. *Health Literacy* will be assessed using three items validated in English and Spanish speaking populations [48]. A *Transportation Survey* will assess barriers to visiting health care providers or a pharmacy to obtain medication refills (currently undergoing validation). *Interpersonal Processes of Care*, including communication and other experiences with doctors and staff members, will be assessed by the Interpersonal Processes of Care Survey: Short Form (IPC-18).

Intermediate variables will be collected at four time points (0, 6-, 12-, and 24-months). *Diabetes Knowledge* will be assessed using the Spoken Knowledge in Low Literacy in Diabetes Scale (SKILL-D) (reliability .72) [49]. *Depression* will be measured using the 2-item Patient Health Questionnaire (PHQ-2). *Social Support* will be measured using a 4-item assessment of amount of total support received and satisfaction of support from family, friends and healthcare team [50]. *Autonomous Self-Regulation* will be measured using 6-item Treatment Self-Regulation Questionnaire (TSRQ, reliability 0.85-0.93) [51,52]. The items begin with the stem, “The reason I would take my diabetes and cholesterol medications exactly as prescribed is…” Responses include ratings of reasons for taking medications, such as, “…because I feel personally satisfied when I keep my diabetes and cholesterol within strict guidelines.” *Perceived Competence* will be measured using the 4-item Perceived Competence Scale (PCS) to assess patients’ experiences of feeling able to manage their diabetes successfully (reliability 0.80-0.94) [51,53,54]. Scores are associated with quality of life, medication adherence, and A1c [51,53].

*HP Activity* will be collected on a standardized worksheet completed after every participant contact (by phone and in-person). Information obtained will include mode, time, and content of contact, results of glucose or blood pressure self-monitoring, goals, and interventions. Additional information collected includes *PCP and Pharmacist Activity and Medication Changes.* The reconciled medication list from the medical chart will be used as data to evaluate medication changes and overall complexity of therapy. Intensification of therapy will be defined as the number of increases in the dosage of an antihypertensive agent, hypoglycemic agent, or insulin, or the addition of a new agent since the baseline visit [21]. Chart review will define the number of PCP and pharmacist encounters as well as the number of pharmacist- or physician-initiated medication changes. Chart review will also include information on actual medications prescribed for diabetes, hypertension and hyperlipidemia, co-morbid conditions, and diabetic complications.

*Clinical Outcomes* will be collected at four time points (0, 6-, 12-, and 24-months). Professional research staff will perform phlebotomy and measure blood pressure, weight and height. These staff will be blinded to participant group assignment. Hemoglobin A1c will be obtained via phlebotomy. The laboratory test has National Glycohemoglobin Standardization Program certification. Fasting lipid profiles will also be obtained via phlebotomy, including HDL, LDL, and triglyceride measurements. Both A1c and lipid profile results will be placed in the participant’s EMR. Height and weight measurements will be obtained to determine body mass index (BMI). A calibrated digital scale will measure weight. A height stadiometer will measure body height, with participants removing their shoes. Blood pressure measurements will be recorded on participants sitting down for at least five minutes, following standard procedure.

*Self-Care Behaviors* related to diabetes self-management including diet and physical activity will be evaluated through the Summary of Diabetes Self-Care Activities Measure (SDSCA) [55]. This includes 11 core items on diet, exercise, blood sugar testing, foot care, and smoking. *Medication Adherence* will be quantified using two methods. The Morisky Medication Adherence Scale will be used to assess self-reported adherence for all participants. The 8-item scale (reliability 0.83) will be completed once for adherence to diabetes medication and once for hypertensive medication, if applicable [56]. The second method of adherence evaluation will consist of obtaining objective pharmacy refill data. Each participant will provide contact information for all of the pharmacies where prescriptions have been filled for the past year. The HIPAA authorization form allows research personnel to contact the pharmacies for fill information. The personnel will contact these pharmacies to collect information on the prescriptions filled. For each prescription, the medication name, date dispensed, dosage, frequency, quantity, and days supply will be requested following an approach similar to that taken in a study on asthma [57]. The Proportion of Days Covered (PDC), a measure of adherence, will be calculated for all diabetes and hypertensive medications using the prescription fill data obtained from pharmacies. *Health Related Quality of Life* will be measured using the Diabetes Distress Scale (DDS4) [58], a 4-item measure of disease specific quality of life.

### Cost and cost-effectiveness evaluation

We will conduct a cost-utility analysis comparing the *Pharmacist* and *Pharmacist+HP* conditions, following the guidelines for conducting pharmacoeconomic analyses issued by the Panel on Cost-Effectiveness and Medicine and the International Society for Pharmacoeconomics and Outcomes Research [59-61]. We will conduct the analysis from the *health-system perspective*, taking into account *direct program costs and direct non-program costs.* Direct program costs will include personnel, educational materials, and any visit-related costs. Direct non-program costs will include cost for all major health care utilization for hospital admissions, emergency room visits, outpatient visits and prescription medications during the intervention period. Participants will complete an interview regarding healthcare utilization outside of UIMC during scheduled data collections including emergency room visits and hospitalizations. For health care use received at UIMC, hospital electronic records and billing data for enrolled participants will be used to identify ambulatory and hospital use, professional and technical visit fees, emergency room visits, as well as laboratory and other ancillary services use. Cost estimates for the program costs (salaries for HPs, etc.) will be based on prevailing costs from appropriate published national sources, where possible. For example, healthcare use costs will be estimated using national Medicare reimbursement rates (average DRG rates) for hospitalization, Medicare fee schedules for physician and other professional services, and Medicare’s Resource-Based Relative Value Scale (RBRVS) for outpatient procedures. Since our study will span a time period exceeding one year, costs will be discounted using a rate of 5% per year.

Our effectiveness measures will include improvements in A1c, blood pressure, cholesterol levels and projected quality-adjusted life-years (QALYs). The incremental cost-effectiveness ratio (e.g., cost per change in A1c) of *Pharmacist+HP* vs. *Pharmacist* alone will be estimated at the end of 12 and 24 months. Cost per QALY will be determined from projections of continued *Pharmacist+HP* involvement and assumptions concerning the continued effects of *Pharmacist+HP* on outcomes over time. The QALY and utility estimates will be based on a Markov model (CDC Diabetes Cost-effectiveness Group) [62]. Sensitivity analyses will estimate the impact of changes in factors such as age, induced health-care visits, incidence of complications, HP costs, physician time, and discount rates.

## Discussion

To our knowledge, this will be the first study to evaluate the impact of HPs as a complement to clinical pharmacist management in a randomized trial. The study incorporates evidence-based procedures of clinical pharmacists and community HPs to assess their complementary involvement in diabetes management.

The study design includes clinic-based pharmacists. We chose to study pharmacists because the strongest empirical support for improved diabetes medication management currently lies in PCP-directed collaborative teams with pharmacists. In fact, collaborative medication therapy management by pharmacists has been approved in 46 states in the U.S. since 2008 for diabetes and dyslipidemia [63,64]. This growing level of pharmacist involvement supports the need for further study of pharmacists in team management as part of a patient-centered medical home [65].

To complement pharmacist efforts in reaching minority populations, we include HPs. Despite the endorsement of peer HPs by the American Association of Diabetes Educators (AADE) and American Public Health Association (APHA), they remain controversial in the U.S. healthcare system [66-68]. Criticisms of HPs focus upon the lack of formal evaluation studies supporting their effectiveness on clinical endpoints and weak connections between HPs and providers. This study may provide stronger evidence related to the benefits of HPs when they are closely connected to clinic-based pharmacists.

While a number of studies have examined the impact of HPs on diabetes management, there has been a greater emphasis on lifestyle modification than on medication management. Pharmacists provide a unique resource for HPs with their expertise in medication reconciliation, strategies to improve adherence, side effects, and prescription fill management. HPs may be less comfortable with the complexities of polypharmacy, adjustments of medication dosages, and the evolving nature of novel medication therapies utilized in diabetes care. This research will help in understanding the support that pharmacists may provide to community HPs. Important process data include the means by which pharmacists and HPs communicate (by telephone, in-person, or secure electronic mail).

A number of challenges and concerns have been considered. First, there may be significant variation in HP attention and impact. To address this, health promoters will be uniformly trained through a CDC-funded curriculum. We include regular re-evaluation of activities to detect drift. Health promoters will follow an established protocol for patient contact; and will maintain a log to be submitted to a supervisor for evaluation. Next, other health care providers, including PCPs, may adjust therapy independent of study procedures. Medical record reviews will describe medication changes if completed within the UIMC health care system (where it is expected to most commonly occur). Analyses will include medication changes by pharmacist or other provider. Finally, while a majority of Latino patients are Mexican-American in Chicago (~75%), others come from Puerto Rico or elsewhere, and may have a different cultural background. Health promoters are trained to be sensitive to these differences, in cases where there may be a mismatch between health promoter and client.

In conclusion, the study will evaluate an innovative, integrated approach to chronic disease self-management in minorities with poorly controlled diabetes. The approach is comprised of clinic-based pharmacists and community-based health promoters collaborating together. Pharmacists and HPs will target patient-level factors (i.e., lack of adherence to lifestyle modification and medications) and provider-level factors (i.e., clinical inertia) that contribute to poor clinical outcomes in diabetes. Importantly, the study design and analytic approach will help determine the differential and combined impact of adherence to lifestyle changes, medication, and intensification on clinical outcomes.

## Abbreviations

AADE: American Association of Diabetes Educators; APHA: American Public Health Association; BMI: Body Mass Index; DDS: Diabetes Distress Scale; DEEP: Diabetes Empowerment Education Program; DRG: Diagnosis Related Group; EMR: Electronic Medical Record; FQHC: Federally Qualified Health Centers; HIPAA: Health Insurance Portability and Accountability Act; HP: Health Promoter; ICC: Intraclass Correlation Coefficient; MTM: Medication Therapy Management; PCP: Primary Care Physician; PDC: Proportion of Days Covered; PCS: Perceived Competence Scale; QALY: Quality Adjusted Life Year; RA: Research Assistant; RBRVS: Resource-Based Relative Value Scale; SDSCA: Summary of Diabetes Self-Care Activities Measure; TSRQ: Treatment Self-Regulation Questionnaire; UIMC: University of Illinois Medical Center.

## Competing interests

The authors declare that they have no competing interests.

## Authors’ contributions

BG and LS conceived of the study, and participated in its design and coordination, and drafted the manuscript. LS provided overall study coordination, staff training, and manuscript revision. AC assisted in community health worker training protocol, and JT assisted in pharmacist protocol. DT, DM, and MB participated in study design and analytic plan. All authors read and approved the final manuscript.

## Pre-publication history

The pre-publication history for this paper can be accessed here:

http://www.biomedcentral.com/1471-2458/12/891/prepub
